# Prescription of pain medication among older cancer patients with and without an intellectual disability: a national register study

**DOI:** 10.1186/s12885-019-6290-0

**Published:** 2019-11-04

**Authors:** Mikael Segerlantz, Anna Axmon, Rebecca Gagnemo Persson, Eva Brun, Gerd Ahlström

**Affiliations:** 1 Department of Clinical Sciences, Oncology and Pathology, Institute for Palliative Care, Faculty of Medicine, Lund University, Lund, Sweden; 2 Department of Palliative Care and Advanced Home Health Care, Primary Health Care Skane, Region Skane, Lund, Sweden; 3 EPI@LUND (Epidemiology, Population studies, and Infrastructures at Lund University), Division of Occupational and Environmental Medicine, Lund University, Lund, Sweden; 4 Department of Health Sciences, Faculty of Medicine, Lund University, Box 157, 221 00 Lund, Sweden; 5 Department of Clinical Sciences, Oncology and Pathology, Faculty of Medicine, Lund University, Lund, Sweden; 6 Department of Radiotherapy and Radiophysics, Skane University Hospital, Lund, Sweden

**Keywords:** Cognitive impairment, Mental retardation, Learning disability, Medication, Neoplasms, Pain

## Abstract

**Background:**

The longevity for people with intellectual disability (ID) has significantly increased in developed countries during the past decades. Consequently, the incidence of cancer is expected to increase in this group. The aim of the present study was to investigate the prescription of pain medication in older cancer patients with intellectual disability (ID) compared to older patients in the general population, surviving or living with a cancer diagnosis.

**Methods:**

This Swedish national registry-based study, included people with ID aged 55 years or older in 2012, and alive at the end of that year (ID cohort, *n* = 7936). For comparisons, we used a referent cohort, one-to-one matched with the general population by year of birth and sex (gPop cohort, n = 7936). People with at least one diagnosis of cancer during 2002–2012 were identified using the Swedish National Patient Register, resulting in 555 cancer patients with ID and 877 cancer patients from the general population. These two cohorts of cancer patients were compared with respect to prescription of pain medication for the period 2006–2012. Outcome data were aggregated so that each patient was categorized as either having or not having at least one prescription of each investigated drug group during the study period, and relative risks (RRs) for prescription were estimated for prescription in the ID cohort vs the gPop cohort.

**Results:**

Cancer patients with ID were less likely than cancer patients in the gPop cohort to have at least one prescription of COX inhibitors (RR 0.61) and weak opioids (RR 0.63). They were, however, more likely to be prescribed paracetamol (RR 1.16), antidepressants (RR 2.09), anxiolytics (RR 2.84), and “other hypnotics, sedatives, and neuroleptics” (RR 1.39). No statistically significant differences between the two cohorts were found for strong opioids, antiepileptics, tricyclic antidepressants, or hypnotics and sedatives.

**Conclusion:**

In the studied cohort of older people surviving or living with cancer, prescriptions for pain-treatment was less common in patients with ID compared to the general population. These results may suggest that pain is not sufficiently treated among cancer patients with ID, a situation that most likely would compromise the quality of life in this group.

## Background

### Cancer pain and symptom assessment

Pain is a common symptom in cancer and thus a major problem. About one-third of cancer patients have pain at the time of diagnosis and two-thirds in the advanced stage of their disease [1, 2]. In addition to pain, patients with advanced cancer often suffer from symptoms such as fatigue, weakness, dyspnea, nausea, constipation and impaired cognitive ability, which further magnifies the impact of pain and impairs quality of life [2, 3]. Untreated or inadequately treated pain increases depression rates, [1] compromises sleep and appetite, and may increase the cognitive dysfunction in older persons [4] and thus be a major hindrance to the maintenance of functional independence [5]. Furthermore, poorly managed pain in older and frail patients increases the needs of and costs for healthcare [6, 7].

Symptom assessment and the intensity of pain is preferably self-reported, measured using numerical rating scales [8]. Self-reporting of pain is more challenging but still feasible in non-verbal situations or in cognitively impaired older persons [9–11]. The choice of appropriate analgesic therapy should be carefully made based on the magnitude and character of pain, patient co-morbidity and compliance to prescriptions. An important aspect in achieving adequate pain control and minimize the risk for adverse effects, in older and cognitively impaired people, is an appropriate and recurrent assessment of pain, careful opioid titration, and management of the numbers and types of drugs prescribed at the same time [12, 13].

### Cancer pain in people with an intellectual disability

The longevity for people with intellectual disability (ID) has significantly increased in developed countries due to better living conditions and medical advances [14]. Consequently, the incidence of cancer is expected to increase in this group. Studies on cancer incidence, [15–17] and cancer mortality [18–21] suggest that all ages together, cancer is equally common in people with ID as in the general population. The complexity of pain and the difficulty for people with ID to describe and verbalize their health problems will directly affect how their pain symptoms are perceived and interpreted by caregivers and healthcare professionals [22, 23]. Unfortunately, this may lead to misunderstanding by the healthcare professionals unaccustomed to dealing with people with ID [24, 25]. Frequently, behavioral changes are seen which may be misunderstood as symptoms related to their ID rather than to an underlying physical suffering with pain. This poses a challenge for healthcare professionals to accurately identify the grounds for the behavior and complaints. A recent survey also found that health care professionals experienced difficulties in recognizing, assessing, and treating cancer pain among people with ID [26]. Cancer pain in people with ID is a sparsely investigated topic and is explained by the inherent difficulties regarding the complex interplay of comorbidities and communication problem in people with ID [26]. As pain is an important sign of disease-progression in cancer, miscommunication, and misunderstandings of symptoms can have serious consequences in delaying the diagnosis and proper treatment of the underlying cancer [25]. Given these obstacles, there is a need to explore how pain is managed in the older cancer population with ID.

## Aim

The aim of the present study was to investigate the prescription of pain medication in older cancer patients with ID compared to older patients in the general population, surviving or living with a cancer diagnosis.

## Methods

This was a register-based study, using Swedish national registers both to identify the study cohorts and to collect data on cancer diagnoses and drug prescriptions.

*Swedish national registers used in the current study*The **LSS register** [27] covers support and services provided by all 290 municipalities in Sweden, according to the criteria in the Swedish Act Concerning Support and Service for Persons with Certain Functional Impairments (Swedish abbreviation: LSS law). The LSS Act gives people with significant and permanent functional impairments or disabilities the right to receive special support and services with the purpose of providing them with equal living conditions as those without these disabilities. People receiving such support are classified into three groups, whereof group one comprises people with ID and/or with autism spectrum disorders (ASD). The LSS services and support available according to the law are eight for adult people e.g. group home, occupation at daily living centers, companion service, relief service in home, personal assistants etc. No diagnosis is registered in the LSS register only group code of disability.

National data on inpatient and outpatient specialist healthcare visits are collected in the **National Patient Register** [28]. One primary and up to 21 secondary diagnoses are recorded, coded according to the International Statistical Classification of Diseases and Related Health Problems 10th Revision (ICD-10). The primary diagnosis represents the specific cause of the healthcare visit, as determined by the end of the visit, whereas the secondary diagnoses represent health issues of importance for the diagnosis and/or for the actual treatment of the primary diagnosis. Ongoing healthcare at the end of the study period (year 2012) is not included in the study as all registrations are made at the date of discharge from the healthcare visit.

All dispensed prescribed medication in Sweden, corresponding to 84% of all drugs sold, [29] is recorded in the **Swedish Prescribed Drug Register (PDR)**, which was established in July 2005. Prescribed drugs are registered coded according to the Anatomic Therapeutic Chemical (ATC) classification system at the time of dispensation.

The Swedish National Board of Health and Welfare is the register holder for these three registers.

The **Swedish Population Register** contains data on all persons living in Sweden and includes data as name, age, gender, current residential address, and place of birth. Statistics Sweden is the responsible authority for this register, which is an extract of the census kept at the national tax offices.

### Study cohorts

Through the LSS register, we identified all people with at least one registered measure of service and support during 2012, aged at least 55 years and alive at the end of that year. These comprised the ID cohort (*n* = 7936). Statistics Sweden provided us with a cohort of people from the general population (gPop cohort), one-to-one matched by sex and year of birth.

We used data from the NPR for the period 2002–2012 to identify cancer diagnoses (ICD-10 diagnoses C00-C97) during this time-period for these two cohorts. At least one diagnosis of cancer was found for 555 (7%) people in the ID cohort and 877 (11%) people in the gPop cohort [30].

### Drug prescription

Through the PDR, we collected information on prescribed dispensed drugs for treatment of pain during 2006–2012. The drugs were aggregated into **COX inhibitors** (i.e. NSAIDs) excluding COX2 inhibitors and glucosamine, **paracetamols**, **strong opioids**, **weak opioids**, **antiepileptics** used for treatment of pain, and **tricyclic antidepressants** used for treatment of pain (Table 1). We also assessed **antidepressants**, **anxiolytics**, **hypnotics and sedatives**, and “**other hypnotics, sedatives, and neuroleptics**”.

**Table 1:** Drug classes investigated in the present study

Drug Class	Generics	Anatomical TherapeuticChemical (ATC) Classification
COX-inhibitors	NSAIDs	M01A
Paracetamols	Paracetamol	N02BE01, N02BE51, N02BE71
Opioids (strong)	Morphine	N02AA01, N02AA51, N02AG01
	Oxycodone	N02AA05, N02AJ17–19
	Ketobemidone	N02AB01
	Pethidine	N02AB02
	Fentanyl	N02AB03
	Buprenorphine	N02AE01
	Tapentadol	N02AX06
Opioids (weak)	Codeine	N02AJ06–09 N02AA59 N02AA79
	Dextropropoxyphene	N02 AC04
	Tramadol	N02AX02 N02AJ13, N02AJ15
Antiepileptics	Gabapentin	N03AX12
	Pregabalin	N03AX16
	Lamotrigine	N03AX09
	Topiramate	N03AX11
Tricyclic antidepressants	Amitriptyline	N06AA09
	Nortriptyline	N06AA10
Antidepressants	Mirtazapine	N06AX11
	Citalopram	N06AB04
	Escitalopram	N06AB10
	Fluoxetine	N06AB03
	Venlafaxine	N06AX16
	Sertraline	N06AB06
Anxiolytics	Diazepam	N05BA01
	Oxazepam	N05BA04
	Lorazepam	N05BA06
	Alprazolam	N05BA12
Hypnotics and sedatives	Nitrazepam	N05CD02
	Flunitrazepam	N05CD03
	Midazolam	N05CD08
Other hypnotics, sedatives, and neuroleptics	Haloperidol	N05 AD01
	Clomethiazole	N05CM02
	Zopiclone	N05CF01
	Zolpidem	N05CF02
	Propiomazine	N05CM06
	Hydroxyzine	N05BB01
	Risperidone	N05AX08
	Levomepromazine	N05AA02

### Potential confounding

Using the NPR for 2002–2012, we identified people who had at least one diagnosis of pain (G43-G44, R51, M00-M25, M40-M54, M75-M79, R00-R19, or R30-R29 in ICD-10) during this time. In addition, as some of the pharmaceuticals investigated have other main indicators for prescription, we identified those with at least one diagnosis of epilepsy (G40 and G41 in ICD-10) or depression (F32 and F33).

### Statistics

Analyses of dichotomous outcomes (e.g. having at least one prescription) were performed using generalized linear models (GLM), estimating relative risks (RRs) with 95% confidence intervals (CIs). Analyses were adjusted for diagnosis of pain, and for epilepsy and depression, when appropriate (e.g. diagnosis of epilepsy was adjusted for when investigating prescription of antiepileptic).

All analyses were performed using IBM SPSS Statistics version 25.0 (International Business Machines Corporation (IBM), Armonk, NY, USA). Analyses were only performed if the two groups to be compared comprised at least five observations. A two-sided *p*-value below 0.05 was considered statistically significant.

## Results

Cancer patients with ID were almost three times more likely than those in the gPop cohort to have at least one prescription of anxiolytics and more than twice as likely to have a prescription of antidepressants (Table 2). The latter effect remained after adjustment for the diagnosis of depression. Increased prescription for cancer patients with ID was also found for “other hypnotics, sedatives, and neuroleptics” and paracetamol. Decreased prescription for cancer patients with ID was found for COX inhibitors and weak opioids (Table 2). Adjusting for diagnoses of pain, epilepsy, and depression, respectively, did not change the results.

**Table 2:** Number of people with prescription of pain medication in a cohort of people with intellectual disability (ID) and cancer (*n* = 555) and a referent cohort of people with cancer in the general population (gPop; *n* = 877)

	gPop	ID	ID vs gPop	
	n (%)	n (%)	Crude RR (95% CI)	Adj RR (95% CI)
COX inhibitors	522 (60)	201 (36)	**0.61** (0.54–0.69)	**0.64** (0.56–0.72)^a^
Paracetamol	475 (54)	350 (63)	**1.16** (1.07–1.27)	**1.22** (1.12–1.33)^a^
Strong opioids	171 (19)	105 (19)	0.97 (0.78–1.21)	1.08 (0.87–1.34)^a^
Weak opioids	371 (42)	147 (26)	**0.63** (0.53–0.73)	**0.68** (0.58–0.79)^a^
Antiepileptics	45 (5)	41 (7)	1.44 (0.96–2.17)	0.99 (0.63–1.55)^b^
Tricyclic antidepressants	46 (5)	19 (3)	0.65 (0.39–1.10)	0.71 (0.42–1.21)^c^
Antidepressants	152 (17)	201 (36)	**2.09** (1.74–2.51)	**1.96** (1.65–2.34)^d^
Anxiolytics	144 (16)	259 (47)	**2.84** (2.39–3.38)	
Hypnotics and sedatives	24 (3)	24 (4)	1.58 (0.91–2.76)	
Other drugs	295 (34)	260 (47)	**1.39** (1.23–1.58)	

*RR* relative risk, *CI* confidence interval

^a^Adjusted for diagnosis of pain; ^b^Adjusted for diagnosis of pain and diagnosis of epilepsy; ^c^Adjusted for diagnosis of pain and diagnosis of depression; ^d^Adjusted for diagnosis of depression

RR in bold is a significant result

## Discussion

### Pain medication among patients with ID

Older cancer patients with ID were less likely to receive prescriptions for pain medications compared with their age-peers in the general population. This is in concordance with previous studies where we have reported that in general [31, 32] as well as among those with a diagnosis of pain, [33] older people with ID are less likely than their counterparts in the general population to be prescribed medication for pain. Furthermore, in the present study, the prescription-pattern differed between the cohorts. Cancer patients with ID were more often prescribed paracetamol and less often COX inhibitors and, weak opioids which was consistent even when adjustments were made for diagnos of pain.. Paracetamol is an effective agent for the management of non-malignant pain and pain caused by cancer. It is not associated with any adverse effects when the maximum recommended doses are not exceeded [34]. Physicians might be reluctant to add or switch to more potent drugs, such as COX-inhibitors and weak opioids, with a potentially higher risk of side-effects. These drugs require more thorough monitoring of possible side-effects, which might be more challenging amongst patients with ID where communication-skills could be compromised. Additionally, comorbidity, such as cardiovascular disease and renal insufficiency, can severely affect toxicity from pain medication [35]. Morbidity burden and multi-morbidity are higher amongst adults with ID and more common in all age groups than in the general population [36]. Consequently, it could be expected that polypharmacy is more prevalent in the ID group and thus drug-interactions are more likely to occur. However, further research is needed to study if people with ID are prescribed paracetamol rather than other pain medication due to the physicians trying to avoid polypharmacy and its negative interaction effects, or if there are other explanations for this prescription.

Furthermore, in our study, cancer patients in the ID cohort were more likely to be prescribed anxiolytics and antidepressants than those in the gPop cohort. Some of these medications are being prescribed for challenging behaviour either due to undiagnosed mental illness or poorly recognized pain [37, 38]. Previous studies have shown that people with ID more often have a diagnosis of mental disorders than the general population [36, 39, 40]. Yet, we found that cancer patients with ID were more likely than those without ID to be prescribed anxiolytics and antidepressants, the latter even after adjustment for diagnosis of depression. This can be attributed to how pain is perceived and communicated to and understood by, caregivers and healthcare professionals. This needs to be looked at more closely.

Symptom-assessment is hampered in patients with ID due to communication difficulties. Low-level reports of pain are perhaps due to communication challenges rather than absence of pain and the misconception that people with ID are insensitive to pain or considered to have a higher pain threshold [41]. Interpretation of symptoms and monitoring of optimal drug dose, drug-drug-interactions and thus possible side-effects is crucial for optimal pharmacological treatment and symptom control. This is especially important during end-of-life care when altered pharmacokinetics and pharmacodynamics may make the adverse consequences more serious in a population of older, multi-morbid patients with ID. The high usage of anxiolytics is a cause of some concern, inasmuch as older and/or frail people are more sensitive to adverse effects of these drugs, and thus needs a close monitoring [42, 43].

### Strengths and weaknesses

In the present study, we used the NPR to identify cancers in both the ID and the gPop cohort. This register contains all diagnoses made in inpatient and outpatient specialist care in Sweden. As cancer diagnoses are rarely made in primary care only, we are likely to have identified all cases in the two cohorts. Even if the timeframe was restricted (2002–2012), the material consisted of a large cohort of 7936 older people with ID, whereof 555 with a cancer diagnosis.

Unfortunately, as both the diagnostic data and the data on drug prescription are limited to a defined time period, we are not able to determine the date of the first diagnosis or the first prescription. Therefore, it would be interesting in future research to study the prescriptions before diagnosis and how prescriptions change over the course of cancer.

The included ID cohort having received services and support for people with ID i.e. we considered as a proxy of ID and/or ASD. As the LSS register does not include any information on any diagnoses, this approach may have caused us to include people with ASD but without ID in the ID cohort. It has been approximated that during the period 2004–2010, the group of people with ASD receiving LSS support was about half the size of the group of people with ID receiving the same support [44]. However, older people are less likely to have been diagnosed with ASD, and thus the fraction of people with ASD should be smaller in the present study. Furthermore, ASD often co-occurs with ID [45]. Thus, the influence of people with ASD but without ID should be minor in the present study.

Another weakness with this data source may be that people without LSS support, i.e. those with milder ID or with informal caregivers (e.g. parents or other relatives) only, are not included. However, several facts speak against such a selection. First, all people in the ID cohort were born before the LSS act was passed in 1993. While the LSS act states that people themselves have to apply for support, prior to 1993 people with ID diagnoses were more or less automatically registered for service and support. Having been so, they would continue to receive support even after the introduction of the LSS act. Second, the Swedish welfare system is constructed so that help is provided by the society rather than informal caregivers. Thus, it is unlikely that a person with ID would not have any type of service and support by the municipality. Thirdly, considering the age group studied, the number of people with parents alive and sufficiently healthy to take care of an adult child with ID, without any help from the municipality, is suspected to be small.

Since the study-cohorts consist of people that have survived or are living with their cancer, thus excluding cancer diagnosis with a more dismal prognosis, the distribution of cancer diagnoses might be distorted and the true need for pain medication in patients with cancer and ID is most likely not reflected in our results. However, as the same is true for the gPop cohort (i.e. this too is a cohort of survivors), the cohort comparisons should not have been affected by the exclusion of more severe diagnoses.

The Swedish PDR comprises data on all prescribed drugs dispensed at all pharmacies in Sweden. However, this register does not contain information about over-the-counter-drugs or drugs provided to patients in inpatient care. Among the drugs reported in the present study, only paracetamol can be purchased without a prescription, and the interference of over-the-counter purchases should, therefore, be minor.

## Conclusions

In the studied cohort of older people surviving or living with cancer, prescriptions for pain-treatment were less common in patients with ID compared to the general population. These results may suggest that pain is not sufficiently treated among cancer patients with ID, a situation that most likely would compromise the quality of life in this group. It is important to be sure pain is fully managed for people at all stages, and that behaviour which may be caused by pain, is recognized as pain and treated appropriately. There is a need for further investigations on how pain is assessed and treated in people with ID.

## Data Availability

In order to approve the study, the ethics committee at Lund made restrictions regarding access to the data due to the sensitive information on a very vulnerable group, i.e., persons with ID. Even though the data are anonymized, it contains enough details to enable identification of single individuals. Therefore, the datasets analyzed during the current study are available from the PI (GA) on reasonable request and after approval from the Regional Ethical Review Board. However, as the database is compiled by national register data, other researchers may contact the register holders, the Swedish National Board of Health and Welfare and Statistics Sweden, to get access to the registries used in this study, and thereby generate a similar database.

## Abbreviations

CI: Confidence interval
gPop cohort: cohort of people from the general population
ICD-10: International Statistical Classification of Diseases and Related Health Problems 10th Revision
ID: Intellectual disability
LSS: The Swedish act concerning support and service for people with certain functional impairments
NPR: National Patient Register
PDR: Prescribed Drug Register
RR: Relative risk
